# Supplementary material for: Partial rejuvenation of the spermatogonial stem cell niche after gender-affirming hormone therapy in trans women
Source: eLife. 2025 Jan 7;13:RP94825. doi: 10.7554/eLife.94825 (PMC11706602; doi:10.7554/eLife.94825)
Supplement: Supplementary file 1. [file elife-94825-supp1.docx]

**Supplementary File 1. Reference values for luteinizing hormone.**

| **Sex** | **Age (years)** | **Reference value (U/L)** |
| --- | --- | --- |
| Male | 0 - 1 | 0.10 - 0.43 |
| Male | 1 - 5 | 0.10 - 1.3 |
| Male | 6 - 10 | 0.10 - 1.4 |
| Male | 11 - 12 | 0.11 - 7.8 |
| Male | 13 - 17 | 1.3 - 9.8 |
| Male | Adult | 1.7 - 8.6 |
| Female | Adult | Premenopausal: 1 - 96 |
