# Supplementary material for: Partial rejuvenation of the spermatogonial stem cell niche after gender-affirming hormone therapy in trans women
Source: eLife. 2025 Jan 7;13:RP94825. doi: 10.7554/eLife.94825 (PMC11706602; doi:10.7554/eLife.94825)
Supplement: Supplementary file 2. [file elife-94825-supp2.docx]

**Supplementary File 2. Reference values for follicle-stimulating hormone.**

| **Sex** | **Age (years)** | **Reference value (U/L)** |
| --- | --- | --- |
| Male | 0 - 5 | 0.21 - 2.8 |
| Male | 6 - 10 | 0.37 - 3.8 |
| Male | 11 - 13 | 0.44 - 4.6 |
| Male | 14 - 17 | 1.5 - 12.9 |
| Male | Adult | 1.5 – 12.4 |
| Female | Adult | Premenopausal: 1.7 – 21.5 |
