# Supplementary material for: Partial rejuvenation of the spermatogonial stem cell niche after gender-affirming hormone therapy in trans women
Source: eLife. 2025 Jan 7;13:RP94825. doi: 10.7554/eLife.94825 (PMC11706602; doi:10.7554/eLife.94825)
Supplement: Supplementary file 3. [file elife-94825-supp3.docx]

**Supplementary File 3. Reference values for testosterone.**

| **Sex** | **Age (years)** | **Reference value (ng/dL)** |
| --- | --- | --- |
| Male | 0 - 1 | 3 - 363 |
| Male | 1 - 4 | 3 - 20 |
| Male | 4 - 7 | 3 - 26 |
| Male | 7 - 9 | 3 - 23 |
| Male | 9 - 11 | 2 - 57 |
| Male | 11 - 13 | 7 - 747 |
| Male | 13 - 15 | 33 - 585 |
| Male | 15 - 17 | 185 - 886 |
| Male | Adult | 218 - 929 |
| Female | Adult | Premenopausal: 5.8 - 59 |
