# Supplementary material for: Partial rejuvenation of the spermatogonial stem cell niche after gender-affirming hormone therapy in trans women
Source: eLife. 2025 Jan 7;13:RP94825. doi: 10.7554/eLife.94825 (PMC11706602; doi:10.7554/eLife.94825)
Supplement: Supplementary file 4. [file elife-94825-supp4.docx]

**Supplementary File 4. Reference values for estradiol.**

| **Sex** | **Age (years)** | **Reference value (ng/L)** |
| --- | --- | --- |
| Male | <9 | <6 |
| Male | 9 - 12 | <10 |
| Male | 12 - 15 | 1 - 36 |
| Male | 15 - 17 | 3 - 34 |
| Male | Adult | 8 – 42 |
| Female | Adult | Premenopausal: 30 - 400 |
