# Supplementary material for: Partial rejuvenation of the spermatogonial stem cell niche after gender-affirming hormone therapy in trans women
Source: eLife. 2025 Jan 7;13:RP94825. doi: 10.7554/eLife.94825 (PMC11706602; doi:10.7554/eLife.94825)
Supplement: Supplementary file 6. [file elife-94825-supp6.docx]

**Supplementary File 6. Reference values for Inhibin B.**

| **Sex** | **Tanner stage** | **Reference value (µg/L)** |
| --- | --- | --- |
| Male | G1 | 35 - 182 |
| Male | G2 | 62 - 338 |
| Male | G3 | 78 - 323 |
| Male | G4 | 67 - 304 |
| Male | Adult | 95 - 323 |
| Female | Adult | Premenopausal: <200 |
